# Controversies surrounding continuous deep sedation at the end of life: the parliamentary and societal debates in France

**DOI:** 10.1186/s12910-016-0116-2

**Published:** 2016-06-29

**Authors:** Kasper Raus, Kenneth Chambaere, Sigrid Sterckx

**Affiliations:** Department of Philosophy and Moral Sciences, Ghent University, Blandijnberg 2, 9000 Ghent, Belgium; End-of-Life Care Research Group Vrije Universiteit Brussel (VUB), Ghent University, Laarbeeklaan 103, 1090 Brussels, Belgium

**Keywords:** Continuous deep sedation, Legal developments, France, Ethical analysis

## Abstract

**Background:**

Continuous deep sedation at the end of life is a practice that has been the topic of considerable ethical debate, for example surrounding its perceived similarity or dissimilarity with physician-assisted dying. The practice is generally considered to be legal as a form of symptom control, although this is mostly only assumed. France has passed an amendment to the Public Health Act that would grant certain terminally ill patients an explicit right to continuous deep sedation until they pass away. Such a framework would be unique in the world.

**Discussion:**

In this paper we will highlight and reflect on four relevant aspects and shortcomings of the proposed bill. First, that the bill suggests that continuous deeps sedation should be considered as a sui generis practice. Second, that it requires that sedation should always be accompanied by the withholding of all artificial nutrition and hydration. In the most recently amended version of the legal proposal it is stated that life sustaining treatments are withheld unless the patient objects. Third, that the French bill would not require that the suffering for which continuous deep sedation is initiated is unbearable. Fourth, the question as to whether the proposal should be considered as a way to avoid having to decriminalise euthanasia and/or PAS or, on the contrary, as a veiled way to decriminalise these practices.

**Summary:**

The French proposal to amend the Public Health Act to include a right to continuous deep sedation for some patients is a unique opportunity to clarify the legality of continuous deep sedation as an end-of-life practice. Moreover, it would recognize that the practice of continuous deep sedation raises ethical and legal issues that are different from those raised by symptom control on the one hand and assisted dying on the other hand. Nevertheless, there are still various issues of significant ethical concern in the French legislative proposal.

## Background

### Introduction

Continuous deep sedation at the end of life (CDS) is the practice whereby a physician uses sedative medication resulting in the reduction or elimination of a patient’s consciousness until she dies. This practice is the topic of considerable ethical debate [1–3]. Part of the controversy surrounding continuous sedation is related to its perceived similarity or dissimilarity to euthanasia and/or physician-assisted suicide (PAS) [4]. Some regard the practice as proper medical care [5] whereas others see continuous sedation as equivalent to 'slow euthanasia' [6]. Most commentators agree that continuous sedation at the end of life can be ethically justified in particular circumstances.

Currently the debate on sedation is very lively in France, where an amendment to the Public Health Act was made to include an explicit right for terminally ill patients to 'deep and continuous sedation' [7]. This has received little attention in the academic literature and in the international news media, yet it represents a development that is highly interesting for various reasons. First, if France would indeed create an explicit legal right to obtain continuous deep sedation this would be unique in the world. Continuous deep sedation is deemed legal in most countries, but mostly because it is assumed to be a form of legally allowed symptom control. Second, various elements of the French legislative proposal would create a very strict regulatory framework for sedation. For example, there is considerable debate over whether deep and continuous sedation should always be accompanied by the withholding or withdrawing of all life sustaining treatment, including artificial hydration and nutrition, with the initial proposal stating that it should always be withheld. Moreover, the proposal requires health institutions to create a register documenting all cases of continuous sedation until death occurring on their premises. Such a registration has never before been put into place. Third, it could be claimed that in some respects the French proposition seeks to legalise continuous deep sedation so as to avoid having to decriminalise physician-assisted suicide. If true, this would support the idea that the availability of sedation is sometimes used as an argument not to decriminalise euthanasia or physician-assisted suicide [8] or that discussions on continuous deep sedation distract from important debates on euthanasia or physician assisted suicide and that we are thus perhaps 'pulling the sheet over our eyes' [1].

This paper will elaborate on the current ethical and legal debate in France and on the unique and ground-breaking nature of the legislative proposal on continuous sedation. We will highlight and reflect on four ethically relevant aspects and shortcomings of the bill. First, that the bill suggests that continuous deep sedation should be considered as a *sui generis* practice. Second, that it requires that sedation should normally be accompanied by the withholding of all life sustaining treatment. Whether this also includes artificial nutrition and hydration is, as will be discussed below, a matter of considerable debate. Third, that the French bill would not require that the suffering for which continuous deep sedation is initiated is severe or unbearable. Fourth, the question as to whether the proposal should be considered as a way to avoid having to decriminalise euthanasia and/or PAS or, on the contrary, as a veiled way to decriminalise these practices.

### The legislative developments in France

Patients' rights and end-of-life care in France are regulated by the law of 22 April 2005 [9]—also known as the *Loi Leonetti* (Leonetti Law) after the man who originally proposed the law— which amends the Public Health Act (*Code de la Santé Publique*, CSP). This law explicitly allows physicians to provide far-reaching symptom control, even at the risk of shortening life, while prohibiting physician-assisted suicide and euthanasia. It has been suggested that the *Loi Leonetti* also allows continuous deep sedation as a form of far-reaching symptom control [10], although it does not mention the practice.

The end of life remains a topic of legal, political and ethical debate in France. When running for President in 2012, François Hollande made various commitments, one of which (commitment 21)^1^ was to allow terminally ill patients with unbearable suffering the possibility to 'benefit from medical assistance to end one's life with dignity'.^2^ After being elected President, to follow up on his commitment, François Hollande entrusted Professor Didier Sicard, a physician specialised in internal medicine and a former Chair of the National Advisory Committee on Ethics, with a mission to examine the need to rethink the French legal framework on end-of-life care. This mission resulted in a report (known as the Sicard report) which stated that the *Loi Leonetti* already allowed many practices such as continuous deep sedation, but that this was generally unknown [11]. The same report condemned the practice of euthanasia, while remaining ambiguous on physician-assisted suicide. It was concluded that a proper use of the *Loi Leonetti* could address most requests for life-ending.

Following the Sicard Report, on 21 January 2015, Alain Claeys and Jean Leonetti submitted a legislative proposal to France's National Assembly (i.e., the House of Representatives) to introduce various significant amendments to the existing *Loi Leonetti* which, 10 years after its adoption, was deemed to be unclear in some respects. The proposal was a multi-party proposal, as Claeys is a deputy from the left wing of the political spectrum, while Leonetti belongs to the right wing. Particularly relevant for this paper are two specific changes proposed by Claeys and Leonetti. The first is the amendment to include an explicit legal right to continuous deep sedation. They proposed to include the following statement in the Public Health Act:

*At the request of the patient to avoid all suffering and to not unnecessarily prolong his or her life, a deep and continuous sedation producing a change in consciousness that is maintained until death, associated with pain relief and with the discontinuation of all life-sustaining treatments, is put into place in the following cases:*

1. *when the patient, suffering from a serious and incurable illness, and whose life expectancy is threatened in the short term, shows treatment refractory suffering.*
2. *when the decision of a patient, suffering from a serious and incurable illness, to stop a treatment, threatens his or her life expectancy in the short term* ([7], authors’ translation)^3^

Second, Claeys and Leonetti proposed to include into the Public Health Act the statement that 'artificial hydration and nutrition constitute treatment' ([7], authors’ translation).^4^ This is highly relevant since, combined with the above amendment, this would mean that all deep and continuous sedation would have to be accompanied by the withholding or withdrawing of all artificial food and fluids. Moreover, it would also mean that terminally ill patients who refuse artificial hydration and nutrition could automatically request deep and continuous sedation (see case 2 above).

The proposed amendments have been the topic of considerable debate in the French National Assembly and the French Senate. In a first reading, the legislative proposal was adopted in the National Assembly by a significant majority on 17 March 2015 (436 votes in favour, 34 votes against, 83 abstentions). When the text was subsequently sent to the Senate, unexpectedly, it received significantly less support. Two important amendments were made by the Senate. First, an amendment was adopted to replace the sentence stating that ‘artificial hydration and nutrition constitute treatment’ with the following sentence: 'Artificial hydration constitutes care that can be continued until the end of life'.^5^ Second, an amendment was voted to delete the words 'continuous' and 'maintained until death'. This amendment thus shifted the focus of the bill from the specific case of continuous deep sedation until death to any form of deep sedation (e.g., also intermittent deep sedation or deep sedation that is not maintained until death). As a result of the disagreement between the National Assembly and the Senate, the legislative proposal was rejected by the Senate on 23 June 2015 (196 votes against and 87 in favour) and sent back to the National Assembly.

In a second reading of the bill, the National Assembly ignored the amendments made in the Senate and relied on the initial proposal to focus the bill on continuous deep sedation until death and to consider artificial hydration and nutrition to be treatment. Again, a significant majority approved the legislative proposal. When sent to the Senate for a second reading, the Senate again amended the proposal to state that artificial hydration and nutrition constitute care that can be continued until death. However, this time the Senate approved the bill with a significant majority (10 votes against and 287 in favour), after which the proposal was, in a final stage, discussed in a mixed commission consisting of members of the Assemblée and the French Senate.

In this mixed commission several additional amendments were made, and on 27 January 2016 the proposal became law. The law states that the Public Health Act should be amended to state that: “Artificial hydration and nutrition constitute treatments that can be stopped”.^6^ It also guarantees a right to continuous deep sedation, as the law also amends the Public Health Act to state that:

*At the request of the patient to avoid all suffering and to avoid therapeutic obstinacy a deep and continuous sedation producing a change in consciousness that is maintained until death, associated with pain relief and with the discontinuation of all life-sustaining treatments, is put into place in the following cases:*

1. *when the patient, suffering from a serious and incurable illness, and whose life expectancy is threatened in the short term, shows treatment refractory suffering.*
2. *when the decision of a patient, suffering from a serious and incurable illness, to stop a treatment, threatens his or her life expectancy in the short term and causes unbearable suffering* ([12], authors’ translation)*.*^7^

The text that was passed as law is very similar to the initial proposal by Claeys & Leonetti (quoted above).

Various aspects of the French proposal and the ensuing debates are of particular interest and invite ethical reflections, to which we now turn.

## Discussion

### Is continuous deep sedation a sui generis practice and does it require a separate law?

There is considerable international debate on whether or not continuous deep sedation is a *sui generis* practice. For some, the practice is nothing more than 'slow euthanasia' [6] or 'euthanasia in disguise' [13] and hence should be considered and regulated as such. Others see continuous deep sedation as a far reaching form of symptom control. In a qualitative study into continuous sedation in the UK, Belgium and The Netherlands, for example, not a single UK physician or nurse reported ever using the term 'continuous sedation' or a related term [14]. One UK nurse was quoted as saying: 'I don’t usually use the word ‘sedation’, I use the term ‘make him more comfortable and settled' ([14], p.52). These respondents clearly did not experience continuous sedation as a practice that differs from other forms of symptom control.

By discussing a possible legislative framework specifically pertaining to continuous deep sedation, French politicians are suggesting that continuous deep sedation is a practice that is clearly and relevantly different from both symptom control (which was already regulated by the *Loi Leonetti*) and euthanasia or physician-assisted suicide (which remain illegal in France). In most countries, continuous deep sedation is currently considered to be legally allowed as a far-reaching form of symptom control. This legal permissibility is, however, always assumed rather than argued for, and although many international guidelines exist on sedation, only the Dutch National Guideline [15] has explicit legal authority.^8^ In Belgium, when the euthanasia law was being debated, the Council of State advised Parliament to include into the law a section to distinguish euthanasia from 'sedative therapy'. The Council of State argued that the legal status of sedation would otherwise remain uncertain [16]. This advice was ignored and a chance to settle the issue of the legality of continuous deep sedation in Belgium was missed. Meanwhile, uncertainty and confusion persist concerning the boundaries between continuous deep sedation and euthanasia [17–19].^9^

One of the areas of uncertainty regarding continuous deep sedation has to do with the question as to whether or not the practice shortens life. Although it is sometimes claimed that the practice does not shorten life [20], other commentators disagree [21, 22]. Admittedly, even if it could shorten life, continuous sedation could still be legally justified, for other types of legally allowed symptom control (e.g., high doses of analgesics) or treatment limitation could also potentially shorten life. In such cases, a double effect type of reasoning is often employed, i.e., the reasoning that, although shortening life is possible, this is not intended and merely occurs as a side-effect. This type of reasoning is also often applied in the case of continuous deep sedation, yet its soundness in this context is questionable [23].

Moreover, even if the practice of CDS would not shorten life, it does reduce or take away a patient's consciousness until death, which can constitute a serious harm. In view of this fact, according to some experts in medical law, the legal permissibility of continuous sedation is not self-evident [24]. France could thus become the first country in the world to provide explicit legal clarity in this regard.

Creating a specific legislative framework has the further advantage of recognizing that continuous deep sedation raises considerable ethical issues of its own, and that these issues are different from those raised by symptom control on the one hand and euthanasia and physician-assisted suicide on the other hand. Continuous deep sedation is often portrayed as 'normal medical practice', i.e., no different from other practices. Such a view can serve to obscure and/or minimize some of the ethical issues relating to CDS [21]. However, as noted by some commentators, continuous deep sedation at least involves permanently taking away consciousness, which is a grave act that can only be justified by proportionally grave reasons. Patients receiving continuous deep sedation are no longer able to communicate and express their wishes, and are bereft of all experiences, both positive and negative. This provides a reason, in our view, to not automatically consider continuous deep sedation as normal medical practice and hence justified.

The distinction between continuous deep sedation and other medical end-of-life practices is also highlighted by the fact that the initial proposal to amend the *Loi Leonetti* required every institution to maintain a register of all cases of continuous deep sedation, which would have to respect patient anonymity and, when requested, could be made available to the Regional Health Agency. Such a register would be unique and would provide a possibility to monitor the use of continuous deep sedation in France. In contrast, medical decisions such as withdrawing life-support and administering high doses of analgesics would not have to be reported, indicating that they are perceived differently.

In creating an explicit patient right to continuous deep sedation, France would thus be one of the first countries to recognize the practice as a *sui generis* end-of-life practice that poses specific problems and thus needs specific attention. Moreover, in implementing a register, France would be unique.

### Should artificial nutrition and hydration always be withdrawn or withheld?

In many respects, the French proposal calls to mind recommendations already made by some international guidelines [15, 25, 26] (although the content of the various guidelines regarding continuous sedation differs significantly [27]). Continuous deep sedation would require a patient request, and could only be used for patients with a short life expectancy experiencing refractory symptoms and who have a serious and incurable illness. In one highly relevant aspect, the French proposal differs from many guidelines, namely on the issue of artificial nutrition and hydration (ANH). Not surprisingly, this issue has spurred a great deal of debate and controversy in the French Senate.

As mentioned earlier, the initial proposal by Claeys and Leonetti, that was passed by an overwhelming majority at the stages of the first and second reading in the *Assemblée*, stated that continuous deep sedation would always be accompanied by the withdrawing or withholding of all life sustaining treatments. In an official rapport of the Office parlementaire d’évaluation des choix scientifiques et technologiques (OPECTS), Leonetti remarked:

*When administering sedation in the terminal phase, does it have to be accompanied by a withholding of all life sustaining treatments? Alain Claeys and myself think it does. Because-pardon the boldness of my comparison-you do not at the same time hit the break and the accelerator* ([28], p. 68: authors’ translation).

In view of the fact that the initial proposal explicitly stated that ANH constitutes treatment, it diverges from several sedation guidelines, which emphasise that any decision to withdraw or withhold ANH should be separate from the decision whether or not to sedate. Indeed, according to most guidelines, ANH may be continued if there is a reason to do so (for example, for medical reasons, a request from the patient, etc.) [15]. It has been argued that combining sedation and withholding ANH changes the nature of the act and makes it more problematic from an ethical point of view (see, for example, Holm 2013, [22] who argues that sedation without ANH is often disproportionate for in such cases an intervention is possible that achieves the same effect (symptom relief) while posing less risk of life shortening, namely sedation with ANH). Most likely, the requirement that nutrition and hydration should be withheld serves the purpose of not prolonging life, since the goal of continuous deep sedation as defined in the legislative proposal is to 'not unnecessarily prolong the patient’s life' (see above). However, whether or not ANH is medically futile is not, in our view, settled by reference to an incurable illness, a short life expectancy, and refractory suffering, as the proposal suggests. Instead, the decision not to provide ANH seems to imply the moral claim that life is no longer worth prolonging, rather than the medical claim that hydration and nutrition are medically futile.

As mentioned above, the statement that ANH constitutes treatment was twice amended in the French Senate to become the statement that artificial hydration does not constitute treatment, but instead constitutes care that could be maintained until death. Continuing to provide nutrition and hydration should thus, according to most Senators, not be seen as therapeutic obstinacy. The current proposal, as approved after the second reading in the Senate, states that all life sustaining treatment is withheld by default, unless the patient objects. This means that even if ANH is considered a life sustaining treatment, it could still be administered if the patient objects to its withdrawal. However, in this proposal the decision to stop all life sustaining treatments is still automatically tied to the decision to sedate, and it is left up to the patient to object. If the patient does not object, there is no option for the physician to decide to continue life sustaining treatment should she believe it necessary to do so.

Naturally, the issue of whether or not ANH is a treatment to be withheld or care to be continued has relevance beyond the context of continuous deep sedation. It was also the topic of another recent and fierce debate in France, regarding the situation of Vincent Lambert who was in a permanent vegetative state. Lambert’s wife and several of his brothers wanted to have ANH withdrawn, believing this to be in accordance with his wishes when conscious. Vincent Lambert's parents, one sister and one half-brother, however, believed that ANH had to be maintained. After much legal debate the case was brought before the European Court of Human Rights, which judged that countries are allowed to adopt regulation to withdraw or withhold ANH in certain specified circumstances.

Similar to the debate regarding the Claeys and Leonetti proposal, part of the debate on the Lambert case revolved around the issue of whether or not ANH can be considered to be a treatment. According to Lambert's wife, his physician, and six of his eight brothers and sisters, it is indeed a treatment and its continuation could be considered to be therapeutic obstinacy in view of Lambert's medical condition. For Lambert's parents, however, providing food and fluids represents basic care and continuing ANH should therefore not be considered as therapeutic obstinacy. If they were right, then the withdrawal could not be justified under the *Loi Leonetti*.

As the intense debate surrounding the legislative proposal of Claeys and Leonetti demonstrates, this issue is far from settled. Whether ANH represents care or treatment still divides the French public and its representatives. In relation to continuous deep sedation, we believe it is necessary to separate the decision to stop ANH from the decision to sedate. Moreover, we would submit that seeing ANH as only treatment is as problematic as seeing it as purely care. Labelling ANH as treatment runs the risk of portraying the decision to maintain or withdraw it as a purely medical decision. As some commentators have noted, however, nutrition and hydration are only rarely medically futile [22]. If it keeps a patient alive, it serves that purpose, and 'if withdrawal gives rise to symptoms, then continuation is clearly not futile, because it would prevent those symptoms from occurring' ([22], p.233).

The decision to stop artificial hydration and nutrition often seems to be in accordance with the French proposal’s goal ‘to not unnecessarily prolong the patient’s life’ [7]. It is crucial, however, not to invoke the fact that a patient is deeply and continuously sedated as a reason to withhold or withdraw ANH, since this is a ‘salami-slicing technique’ [29] to justify the latter decision merely by reference to a (sedated) state that one has created oneself. A decision to withhold or withdraw ANH can be perfectly justified in many cases, for example by referring to a patient’s request or her personal and moral convictions, but it remains a decision with a significant ethical dimension.

On the other hand, portraying ANH as care ignores the fact that its administration does involve a medical procedure (e.g., feeding tubes or intravenous administration). If a right to refuse treatments is grounded in a patient's fundamental right to refuse unwanted bodily invasions, this may also extend to ANH. Indeed, many of the procedures for administering ANH are the same as those used for interventions that are uncontroversially classified as 'treatment'. Admittedly, despite similarities in the procedure of administration, ANH is seen by many commentators as different in nature from medical treatments [30]. However, even if this is the case, ANH still involves a bodily invasion and could therefore be inconsistent with a patient’s right to bodily integrity if the patient refuses, or, when no longer competent, has refused ANH in advance.

In our view, the question as to whether ANH constitutes medical treatment or medical care is not the most important one to be addressed, and distracts from the more fundamental issue of whether or not ANH can be justifiably withdrawn or withheld in individual cases and what circumstances might justify such a withdrawing or withholding. We believe ANH can in some cases be justifiably withheld or withdrawn. However, in our view, withholding or withdrawing ANH for continuously sedated patients at the end of life is only rarely justified on medical grounds, and should therefore not be portrayed as a purely medical decision. It should be inspired by and based on the wishes and moral and/or religious convictions of patients and/or their relatives, and for this reason it should always be considered on a case-by-case basis. As rightly noted by geriatrician Gillian Craig:

*Staff who believe strongly that intravenous fluids are inappropriate should not impose their views on (…) relatives who request that a dying patient be given intravenous fluids to prevent dehydration or thirst. To overrule such a request is, in my view, ethically wrong. The only proviso would be if the patient had, when* compos mentis*, specifically said that he/she did not want a drip under any circumstances. No relatives should be forced to watch a loved one die while medical staff insist on withholding hydration. (…) Such an experience is deeply disturbing and could haunt a person forever. Is all this agony worth it for the sake of avoiding a drip? (…) The converse also applies. There will be occasions when the medical staff who are professionally involved would like to use a drip, but a knowledgeable relative requests no intervention. In this situation, the medical team will need to make a carefully balanced judgement as to whether intervention is essential or not. (…) Care must be taken to ensure that the burden of bereavement is not loaded heavily by distress about patient management in the terminal phase* ([31], p. 142–3: references omitted).

Therefore, a requirement that ANH should always be withheld when continuous deep sedation is administered to a patient is overly strict and unjustifiably ignores the ethical component of decisions to stop ANH. A policy that ANH could never be withheld or withdrawn is equally problematic, however, as this would ignore the fact that some people may have justified personal, moral or religious reasons for refusing it.

Moreover, giving patients the possibility to decide whether or not to forego life sustaining treatments and ANH also seems to be in accordance with what was initially stated to be one of the goals of the proposal, namely strengthening patients’ decisional authority [28]. During the legislative process, Leonetti, for example, remarked:

*[the proposal] also [concerns] a right of a patient who is most vulnerable because he is at the end of his life and who has the right to ask not to suffer. If there is no other alternative [than CDS] one can legitimately resort to it* ([28], p. 68: authors’ translation).

In a similar vein, Jean Claude Ameisen, president of the Comité Consultatif National d’Ethique, observed that the new legislation is a response to a particular problem:

*[currently], paradoxically, when a patient is conscious and able to express himself, the request for deep sedation is subject to the authority of the physician’s decision. When a patient is at the end of life or when his treatments are withheld, his request to be able to sleep until death* should impose itself on the carers and not be subject to the authority of the physician ([28], p. 40: authors’ translation and emphasis).

However, if ANH is withdrawn by definition (as was specified in the original proposal) or if life sustaining treatments are withdrawn by default unless the patient objects (as mentioned in the most recent amendment of the proposal), the patient’s decisional authority is in fact, we would argue, quite limited.

### Why ignore the severity or (un)bearability of suffering?

Apart from its stance on continuous deep sedation and ANH, the original French legislative proposal differs significantly from international guidelines in one other respect. Many guidelines on continuous deep sedation recommend that it should only be used in patients who are suffering intolerably from treatment refractory symptoms [15, 26]. Although the French legal proposal states that continuous deep sedation should only be initiated for treatment refractory suffering, it does not require this suffering to be severe or unbearable.

Not explicitly including severity or unbearableness of the suffering as a criterion for receiving continuous deep sedation has serious consequences, since refractoriness does not necessarily presuppose severe or unbearable suffering. We believe there are two important reasons for including severity and unbearableness as a condition for obtaining continuous deep sedation. First, taking away a patient’s consciousness is a grave matter that can only be justified for proportionally grave reasons. We would submit that the presence of treatment refractory suffering does not provide a sufficiently grave reason. This condition can only be met when the suffering is also severe and unbearable. The original French legislative proposal would allow patients with a grave and incurable illness to request and receive continuous deep sedation for suffering which, while treatment refractory, is relatively mild. Granted, in view of the fact that, according to the proposal, continuous deep sedation is only indicated for patients who request it, who suffer from serious and incurable illness, and who have a short life expectancy, requests for deep sedation for mild suffering would most likely be rare. Nevertheless, in legislative documents non-explicit criteria are essentially non-existent and thus non-enforceable criteria. Including a criterion regarding the severity and unbearableness of the suffering would foreclose the possibility of requesting deep sedation for mild and tolerable suffering.

Second, omitting a criterion regarding the severity and unbearableness of the suffering shifts the focus from a patient-centered criterion (bearableness) to a purely physician-centered medical criterion (refractoriness). The danger here is that the patient’s voice and his or her appreciation of the situation might be ignored or undervalued. The current proposal might, also in this respect, overly limit patients’ autonomy by excluding the patient from decision-making on what levels of suffering he or she finds bearable and what he or she is willing to trade-off for pain relief.

However, it seems that refractoriness was initially intended to be a patient centred criterion. When explaining his proposal, Leonetti remarked:

*Who decides whether suffering is refractory? The physician or the patient? I have a slight tendency to think that regarding something so fundamentally subjective, even when we have precise criteria for determining current suffering, it is nevertheless the patient who decides that suffering is refractory* ([28], p. 67: authors’ translation).

However, this does not seem to be in line with the way in which the term ‘refractory’ is commonly used, viz.: not or no longer responsive to currently available treatments. Indeed, while severity and bearableness are subjective and can only be judged by a patient, determining whether medical alternatives are available and, if so, which ones, requires medical knowledge. Hence the physician is best suited to judge refractoriness. Moreover, the fact that the French proposal states that refractoriness can be also judged in patients unable to express their wish, suggests that the proposal sees the patient’s voice as non-essential in determining refractoriness.

Therefore, if the goal is to include the patient’s voice, a strong argument can be made for explicitly including severity or unbearableness of the suffering as a criterion. Such a criterion gives patients the right to decide for themselves how they would balance relief of suffering against, for example, alertness at the end of life or other personal, moral or religious values. Moreover, such a criterion creates an obligation on behalf of a patient requesting continuous deep sedation to convince the treating physicians that the suffering he or she experiences is indeed severe and unbearable, which in turn can enable a patient-physician discussion on levels of suffering and different ways to address these.

### Does the stipulation of a right to continuous deep sedation aim to avoid the decriminalisation of euthanasia?

Another topic of fierce debate that erupted in relation to the French legislative proposal is the relationship between continuous deep sedation on the one hand and euthanasia and physician-assisted suicide on the other hand. The legislative proposal attracted considerable criticism from right-to-die organisations for allegedly using the legalisation of continuous deep sedation as a way to avoid having to decriminalise physician-assisted suicide [32].

This criticism was fuelled by the fact that the legislative proposal was a direct consequence of President Hollande’s promise to legalise ‘medical assistance in dying’ (as mentioned above). Hence, what many had interpreted as a promise to decriminalise physician-assisted suicide (but was actually less specific) they saw as eventually being turned into a proposal of a legal framework for continuous deep sedation, much to the discontentment of those in favour of physician-assisted suicide. CDS, they claimed, was already made legal by the *Loi Leonetti* (as was confirmed in the Sicard report), so they considered the creation of an explicit legal right to CDS as no more than smoke and mirrors to distract from the fact that euthanasia and physician-assisted suicide remain illegal in France.

Three elements need stressing in this regard. First, the legislative proposal would create a novel patient right to obtain continuous deep sedation under specific conditions and would thus go beyond what was already explicitly legal. Second, although interpreted by many as a promise to decriminalise physician-assisted suicide, François Hollande's actual commitment was less specific. In some respects, continuous deep sedation could be interpreted as a form of 'medical assistance to end one's life with dignity', thus fulfilling the commitment. Third, although no proposal to legalise physician-assisted suicide or euthanasia is being considered by the French Parliament, these options have been discussed and debated to some extent. The activities of the Sicard taskforce resulted in an extensive report examining and rejecting the possibility to decriminalise euthanasia and/or physician-assisted suicide. Moreover, the National Advisory Committee on Ethics of France has issued an advice showing that the majority of its members favoured creating an explicit right to request continuous deep sedation, while rejecting the decriminalisation of euthanasia or physician-assisted suicide [33].

While some commentators suggest that a right to continuous deep sedation was proposed in order to avoid having to decriminalise physician-assisted suicide or euthanasia, others believe the legislative proposal is actually a disguised way to allow medically assisted life shortening. During the Senate debates, it became clear that many Senators considered the creation of a legal right to continuous deep sedation as nothing more than a first step on the road to the decriminalisation of physician-assisted suicide or euthanasia. For this reason, continuous deep sedation was labelled a 'Trojan horse' [12].

In some respects, this confirms the claim made by Battin [1] that continuous deep sedation often constitutes an inadequate compromise [34]: for some proponents of assisted dying, continuous deep sedation fails to adequately respect patient autonomy and is not indicated in all cases in which assisted dying might be indicated [35], whereas for some opponents of euthanasia, some forms of continuous deep sedation (for example CDS without ANH) could be considered as potentially crossing the border into ethically dubious or unacceptable practices [22]. In short, by missing the opportunity to create clarity on continuous deep sedation, the French legislative initiative has actually resulted in a further polarisation of the debate between proponents and opponents of euthanasia and physician-assisted suicide.

Debating continuous deep sedation together with medically-assisted dying involves considerable risks. If enacting an explicit right to this practice is indeed a disguised way to decriminalise euthanasia or physician-assisted dying, then it is intellectually dishonest. If, instead, the aim of creating a right to continuous deep sedation is to avoid decriminalising medically-assisted dying, this poses risks of its own. As noted by Battin (2008):

*[O]ur anxiety that [sedation] may be confused with euthanasia encourages us to obscure or sanitize the features both practices share* ([1], p.29).

## Conclusions

France is currently in the midst of a fierce debate on what constitutes a dignified end of life and which practices are acceptable for physicians to perform. At the very core of the debate is the practice of continuous deep sedation. As argued in this paper, what is playing out is a unique opportunity to clarify the legality of continuous deep sedation as an end-of-life practice. Recognizing continuous deep sedation as a *sui generis* practice could remove the need to portray the practice either as symptom control or as a form of euthanasia. This might make it less difficult to focus on the issues that are most relevant and pertinent to continuous deep sedation.

However, there are still various issues of significant ethical concern in the French legislative proposal. Automatically withholding artificial nutrition and hydration is in our view overly strict and unjustifiably equates artificial nutrition and hydration with medical treatment. As argued above, there is also danger in seeing it as care that can never be withdrawn. Focussing only on the question as to whether ANH should be seen as just another treatment or as fundamental basic care seems to ignore the most crucial question, i.e., in what circumstances it may justifiably be withdrawn or withheld.

Second, by not limiting the right to continuous deep sedation to patients with severe or unbearable suffering, the French proposal leaves open the possibility to request continuous deep sedation for mild and bearable suffering. In our view, continuous deep sedation is a far reaching practice that can only be justified by reference to an ethical principle of proportionality. However, this ethical justification can only apply to those cases where suffering is both refractory and very severe or unbearable.

Finally, the debates in France demonstrate that discussing continuous deep sedation often also raises the issues of physician-assisted suicide and euthanasia. Opponents of physician-assisted suicide might see continuous deep sedation as a first step on the slippery slope. Others object to the focus on continuous sedation as an excuse to ignore the most pressing issues at the end of life. Continuous deep sedation then proves to be an unacceptable compromise to both proponents and opponents of euthanasia and/or physician-assisted suicide. Moreover, a failure to recognize sedation as a *sui generis* practice and attempts to portray the practice as either similar or dissimilar from medically assisted dying risks ignoring patients’ actual interests in having a peaceful and dignified end of life.

We believe France’s attempt to reflect on continuous deep sedation as a practice that needs clarification provides an interesting example. Most importantly, continuous deep sedation should not be seen as a catch all solution to ethical issues at the end of life, for it is not the automatically preferable alternative it is sometimes portrayed to be [35]. As mentioned above, in France the fear exists that the attempt to legalise continuous deep sedation is fuelled by a desire to avoid debates about euthanasia and/or physician-assisted suicide. In our view, this would be a misguided motivation to create a legal framework for continuous deep sedation. On the other hand, in view of the lack of clarity regarding the legal status of continuous deep sedation, there seem to be good reasons to reflect about ways to render uniform the acceptable conditions for initiating it. This need not necessarily involve creating a specific law as is being developed in France. In The Netherlands, for example, a widely followed national guideline on sedation exists with potential disciplinary ramifications in case of non-compliance. Regardless of the specific regulatory response that is opted for, reflection and decision-making by professional medical bodies as well as public policymakers about when and how continuous deep sedation is acceptable might provide the necessary clarity where it is now often lacking.

## Abbreviations

ANH: artificial nutrition and hydration
CDS: continuous deep sedation at the end of life
CSP: Code de la Santé Publique
OPECTS: Office parlementaire d’évaluation des choix scientifiques et technologiques
PAS: physician assisted suicide

## Footnotes

1 Hollande’s commitments can still be found online at http://www.ps29.org/IMG/pdf/Projet_FH2012.pdf.

2 Original text: *«à bénéficier d’une assistance médicalisée pour terminer sa vie dans la dignité.»*

3 Original text: «*À la demande du patient d'éviter toute souffrance et de ne pas prolonger inutilement sa vie, une sédation profonde et continue provoquant une altération de la conscience maintenue jusqu'au décès, associée à une analgésie et à l'arrêt de l'ensemble des traitements de maintien en vie », est mise en œuvre dans les cas suivants :*

1. Lorsque le patient atteint d'une affection grave et incurable et dont le pronostic vital est engagé à court terme présente une souffrance réfractaire au traitement;
2. Lorsque la décision du patient atteint d'une affection grave et incurable d'arrêter un traitement engage son pronostic vital à court terme».

4 Original text: «La nutrition et l'hydratation artificielles constituent un traitement».

5 Original text: «L'hydratation artificielle constitue un soin qui peut être maintenu jusqu'en fin de vie».

6 Original text: « La nutrition et l’hydratation artificielles constituent des traitements qui peuvent être arrêtés »

7 Original text : « À la demande du patient d’éviter toute souffrance et de ne pas subir d’obstination déraisonnable, une sédation profonde et continue provoquant une altération de la conscience maintenue jusqu’au décès, associée à une analgésie et à l’arrêt de l’ensemble des traitements de maintien en vie, est mise en œuvre dans les cas suivants :

1. Lorsque le patient atteint d’une affection grave et incurable et dont le pronostic vital est engagé à court terme présente une souffrance réfractaire aux traitements;
2. Lorsque la décision du patient atteint d’une affection grave et incurable d’arrêter un traitement engage son pronostic vital à court terme et est susceptible d’entraîner une souffrance insupportable.

8 The Dutch Public Prosecutor has officially declared that he sees no reason to prosecute a physician performing continuous sedation if the Dutch National guideline is respected.

9 Moreover, this confusion does not only exist in Belgium, but also in The Netherlands [36]. There is no reason to assume that this confusion would be any less in other countries, including countries that have not depenalised euthanasia and/or physician-assisted suicide.
